# A quantitative analysis of monochromaticity in genetic interaction networks

**DOI:** 10.1186/1471-2105-12-S13-S16

**Published:** 2011-11-30

**Authors:** Chien-Hsiang Hsu, Tse-Yi Wang, Hsueh-Ting Chu, Cheng-Yan Kao, Kuang-Chi Chen

**Affiliations:** 1 Graduate Institute of Biomedical Electronics and Bioinformatics, National Taiwan University, Taipei, Taiwan; 2 Institute of Information Science, Academia Sinica, Taipei, Taiwan; 3 Department of Computer Science and Information Engineering, Asia University, Taichung, Taiwan; 4 Department of Computer Science and Information Engineering, National Taiwan University, Taipei, Taiwan; 5 Department of Medical Informatics, Tzu Chi University, Hualien, Taiwan

## Abstract

**Background:**

A genetic interaction refers to the deviation of phenotypes from the expected when perturbing two genes simultaneously. Studying genetic interactions help clarify relationships between genes, such as compensation and masking, and identify gene groups of functional modules. Recently, several genome-scale experiments for measuring quantitative (positive and negative) genetic interactions have been conducted. The results revealed that genes in the same module usually interact with each other in a consistent way (pure positive or negative); this phenomenon was designated as monochromaticity. Monochromaticity might be the underlying principle that can be utilized to unveil the modularity of cellular networks. However, no appropriate quantitative measurement for this phenomenon has been proposed.

**Results:**

In this study, we propose the monochromatic index (MCI), which is able to quantitatively evaluate the monochromaticity of potential functional modules of genes, and the MCI was used to study genetic landscapes in different cellular subsystems. We demonstrated that MCI not only amend the deficiencies of MP-score but also properly incorporate the background effect. The results showed that not only within-complex but also between-complex connections present significant monochromatic tendency. Furthermore, we also found that significantly higher proportion of protein complexes are connected by negative genetic interactions in metabolic network, while transcription and translation system adopts relatively even number of positive and negative genetic interactions to link protein complexes.

**Conclusion:**

In summary, we demonstrate that MCI improves deficiencies suffered by MP-score, and can be used to evaluate monochromaticity in a quantitative manner. In addition, it also helps to unveil features of genetic landscapes in different cellular subsystems. Moreover, MCI can be easily applied to data produced by different types of genetic interaction methodologies such as Synthetic Genetic Array (SGA), and epistatic miniarray profile (E-MAP).

## Introduction

Understanding how genotypes determine phenotypes is one of the most important topics in genetics. The maturation of whole genome sequencing techniques and other large scale genomic analysis tools has provided considerable genomic information of many organisms [1]. However, the relationships between genotypes and phenotypes are still far from being fully understood. Phenotypes and genotypes are not one-to-one corresponded; a phenotype is usually simultaneously determined by several genes. Moreover, compensation and epistasis between genes can further complicate the relation between phenotypes and genotypes [2]. Thus, the complex networks of genetic interactions governing phenotypes cannot be understood just by studying each involved gene individually; instead, a systemic manner is required to illustrate the relationships between phenotypes and genotypes.

Several tools for systematically mapping genetic interactions in *Saccharomyces cerevisia*e have been developed; experiments have been conducted to reveal the structure and functional landscape of part of the complex genetic interaction network [2]. Early researches focused on the identification of synthetic lethal genetic interactions, which revealed tremendous functional relationships between genes, numerous compensatory protein complexes, and parallel pathways [3-5]. For example, a systematic deletion analysis in budding yeast demonstrated that only 20% of genes are required for viability [6], which suggests that a robust network is formed by genetic interactions. Nevertheless, the lack of quantitative measurement of genetic interactions in these researches limited the possibility of exploring the genetic interaction landscape in detail.

Recently, two methodologies for quantitatively screening the genetic interactions in *Saccharomyces cerevisia*e have been widely adopted. One is Synthetic Genetic Array (SGA), which was developed by Tong et al. [7]. In SGA methodology, strains with single mutation of query genes are crossed with single mutant strains of array genes, resulting in double mutants. With an automatic robot system, SGA has been used to investigate genetic interactions in a high-throughput manner. Although SGA was initially used to screen synthetic lethality/sickness [7,8], it was later adopted to measure genetic interactions quantitatively [9,10]. The other methodology, called epistatic miniarray profile (E-MAP), is based on measuring the growth rates of single mutant strains and double mutants [11]. In contrast to the unbalanced numbers of query and array genes in SGA, results of E-MAP form a symmetric matrix containing strengths measured by S scores of each pair of genetic interactions [12].

Both SGA and E-MAP require a quantitative definition of genetic interaction. A genetic interaction between two genes is identified when a double mutant phenotype deviates from the empirically determined phenotype based on the two single mutant strains [6,9,12-15]. Several mathematical models have been proposed to quantify the extent of the phenotype deviation [16]. Among all, the multiplicative model is the most widely adopted [9,10,12,13,17-19]. Under the multiplicative model, the deviation (*ε*) is calculated by:(1)

where *φ_AB_* , *φ_A_* , and *φ_B_* are fitness values (such as growth rate and colony size) relative to wild type of double mutation of gene *A* and *B*, single mutation of gene *A*, and single mutation of gene *B*, respectively. Directly from equation (1), genetic interactions can be divided into two categories, positive interactions and negative interactions. Positive interactions, also known as alleviating interactions, refer to a less severe defect of double mutation than expected; they can be further classified into two subgroups, suppression and masking, according to the relationship between a double mutant and the two corresponding single mutations [13,15,17]. In suppression, the fitness of a double mutant is similar to the healthier one of the two single mutants, indicating that deletion of the second gene rescues the defect caused by mutation of the first genes. In contrast, masking occurs when the fitness of a double mutant is equal to the less healthy one of the corresponding single mutants. As opposed to positive interactions, negative interactions, or aggravating interactions, manifest a more severe-than-expected defect, with synthetic lethality being the most extreme case in which the deletion of two nonessential genes leads to cell death.

Numerous researches have suggested the biological scenarios of positive and negative interactions [4,8,11,13,17]. Negative interactions tend to be identified between genes with similar or compensating functions, whereas genes participating in the same pathway or protein complexes often interact to each other positively. These observations suggest that positive interactions connect genes encoding subunits of protein complexes or engaging in the same pathways, while negative interactions indicate functional compensation or parallel pathways [15].

In addition to types of genetic interactions, relationships between two genes can also be investigated by comparing the proximity of their genetic interaction profiles which is the set of all genetic interactions of one gene [10,13,15,17]. It has been suggested that genes forming protein complexes or functioning in the same pathways would have similar genetic interaction patterns. Several hierarchical clustering algorithms have been proposed based on this idea [20-22], providing effective ways to predict gene functions and illustrate the functional modularity of cells.

By integrating genetic interactions with protein complexes and biochemical pathways, nature of genetic interactions has been further explored. By using a simulation framework known as the flux balance analysis [23] to investigate the yeast metabolic system, Segre et al. ascertained that genetic interaction network can be hierarchically clustered into functional modules which interact monochromatically to each other, a phenomenon he designated as monochromaticity [24]. This feature was also reported in a genome-wide study conducted by Costanzo et al [10]. Monochromaticity within protein complexes was also studied [9]. Baryshnikova et al. defined a measure called monochromatic purity score (MP-score) to determine whether a given protein complex was monochromatic. In their research, the majority of protein complexes were reported to be monochromatic, with 46% being pure positive and 37% being pure negative [9]. Moreover, Michaut et al. inspected monochromaticity by studying biological processes [25]. Only 10% of biological processes, defined by Gene Ontology (GO) annotations are monochromatic. This study also showed that less than 1% of interactions between processes were identified to be monochromatic. In addition, they suggested that protein complexes are responsible for the majority of the observed pattern [25,26].

Although monochromaticity is an interesting feature of genetic interaction networks, only few quantitative measures have been proposed. Evaluation of monochromaticity using MP-score has unveiled interesting features of genetic interactions in protein complexes, but a close scrutiny reveals several deficiencies of this index. Firstly, MP-score is affected dramatically by the imbalanced proportions of positive and negative genetic interactions of the background. When the proportions of the two types of genetic interactions are not comparable in a set of genes, MP-score can misjudge the monochromaticity of protein complexes. Secondly, the threshold for which a protein complex is considered to be monochromatic is chosen heuristically. In the work of Baryshnikova et al., the threshold was set to be 0.5; that is, a protein complex was classified to be monochromatic if its absolute value of MP-score was greater than 0.5. Nevertheless, this choice of the threshold has not been corroborated. Lastly, when applying MP-score to some genetic interaction data, the monochromaticity of protein complexes was not observed.

Therefore, an appropriate measure that can be used to unbiasedly assess the monochromaticity of clusters of genetic interactions is desired. This study proposes a new index called monochromatic index (MCI) which improves the deficiencies of MP-score. MCI not only quantitatively is able to evaluate the monochromaticity of potential functional modules of genes but also is useful to study genetic landscapes in different cellular subsystems. We believe that MCI can serve as the basis to explore complex networks of genetic interactions.

## Results and discussion

**Figure 1:**
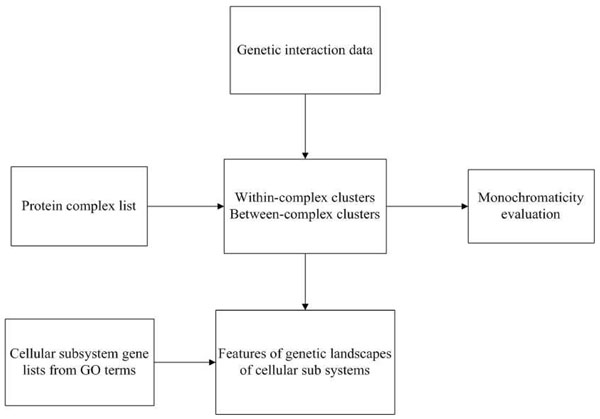
**Overview of work flow.** We download genetic interaction data of Costanzo’s work and classify interactions into within-complex and between-complex clusters using protein complex list provided by Benschop, which is used to evaluate monochromaticity by MCI. Then we assign protein complexes into cellular subsystems they belong and investigate how complexes are coordinated in different cellular subsystems.

We illustrate the work flow with Figure 1. We downloaded the most comprehensive genetic interaction data of *Saccharomyces cerevisiae *[10] and the consensus protein complex list [27]. We divided genetic interactions into two types – within-complex interactions and between-complex interactions, and applied MCI to evaluate the level of monochromaticity for the downloaded data (see Materials and Methods for details). In the following sections, we first show the deficiencies of MP-score, which misjudges the monochromaticity of protein complexes under highly biased background. Then, we demonstrate that MCI incorporates the background information properly to fairly evaluate the significance of monochromaticity not only within but also between protein complexes in the second and the third sections. Our results confirmed that monochromaticity exists in both within-complex and between-complex interactions. Besides, this is the first study, to our knowledge, that shows between-complex interactions present significant degree of monochromaticity. Lastly, with gene lists of different cellular subsystems, MCI was applied to investigate features of genetic landscapes in metabolic as well as transcription and translation systems. We found that metabolic system adopts more negative interactions to coordinate protein complexes, reflecting the robustness of this system. On the other hand, similar amounts of positive and negative interactions between protein complexes were identified in transcription and translation system, suggesting that this system is more vulnerable to protein-complex-dysfunctions. Furthermore, functional relationships between protein complexes within and between modules were also unveiled by MCI-generated networks.

### Deficiencies of MP-score

The goal of this session is to assess, using MP-score, the overall monochromaticity presented by the clusters of within-complex interactions, and judge the performance of MP-score. The MP-score was tested for its applications. We used currently the most comprehensive quantitative genetic interaction data of *Saccharomyces cerevisia*e, generated by SGA [10], to evaluate the monochromaticity of genetic interactions within complexes using MP-score. The data consists of 1,711 query genes crossed to 3,885 array strains, and three different levels of cutoffs were applied, lenient, intermediate, and stringent (Materials and Methods). Yeast protein complexes were adopted from a consensus data set which contains 518 protein complexes obtained by high-throughput predictions and literature curated data [27]. A genetic interaction is classified as a within-complex interaction if the two interacting genes belong to the same protein complex. By this definition, a list of protein complexes and the corresponding within-complex interactions can be constructed. Only complexes containing more than two with-complex interactions were used for further analysis.

We calculated MP-scores for each protein complexes, and the threshold was set as Baryshnikova et al.; that is, complexes with the absolute value of MP-score higher than 0.5 were considered to be monochromatic [9]. Numbers of monochromatic complexes obtained from the three different-cutoff datasets are listed in Table 1. In order to evaluate the significance of monochromaticity of protein complexes, a binomial test was conducted (Materials and Methods). The rationale was that if monochromaticity was not a feature of genetic interactions within complexes, the probabilities of identifying a monochromatic complex and a non-monochromatic complex will be the same, which equals to 0.5. Thus, as the null hypothesis, we assumed that the type, monochromatic or not, of a protein complex follows Bernoulli distribution, with the probability being 0.5. We found that p-values were 2.18e-05, 2.92e-04, and 1.42e-14 for lenient, intermediate, and stringent cutoffs, respectively. The result showed that all of the three cutoffs support that monochromaticity is significant for genetic interactions within protein complexes.

However, we found that the background information was not properly incorporated by MP-score after examining the proportions of positive and negative genetic interactions. Summary of the three different-cutoff datasets is provided in Additional File 1: Table S1 and Additional File 2: Figure S1. These results illustrated that the more stringent the cutoff threshold, was, the more unbalanced the proportions of positive and negative interactions were.

**Table 1:** Number of protein complexes identified in the three data sets.

	Total complex amount	Amount of positively monochromatic complexes	Amount of negatively monochromatic complexes	p-value for observing this proportion
Lenient	76	26	30	2.18e-05
Intermediate	59	18	25	2.92e-04
Stringent	46	18	28	1.42e-14

For each dataset, this table indicates the number of total protein complexes, complexes that were identified to be positively monochromatic by MP-score and complexes that were identified to be negatively monochromatic by MP-score. A complex was defined as monochromatic if its |MP-score|>0.5. The highly biased background (Table S1 and Figure S1) should reduce the significance of monochromaticity of genetic interactions within protein complexes. However, it was not reflected by MP-score.

Especially for the stringent cutoff, about 90% of interactions are negative. This highly biased background should reduce the significance of monochromaticity of genetic interactions within protein complexes. However, it was not reflected by MP-score.

**Figure 2:**
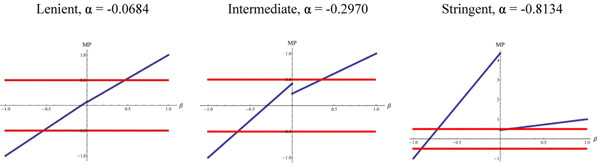
**Effects of background on MP-score.** Plots under different background (α) of MP-score as a function of *β* (see Materials and Mehods). The value *β* is defined as the difference between positive and negative interactions divided by the number of total genetic interactions in a complex. Red lines indicate the threshold above which a protein complex is considered to be monochromatic. Under highly biased background (extreme α value), MP-score is not a proper indicator for monochromaticity.

To investigate the underlying cause, we studied the behavior of MP-score under different α values, which is adjusted to different backgrounds accordingly (Materials and Methods). Figure 2 shows that MP-score becomes more erratic as α deviates from zero. Due to the functional design, the discontinuity of MP-score near zeros becomes more severe when α deviates from zero, which results in an unreasonable situation — considering two complexes, both complexes contain eleven genetic interactions. One of them has six negative interactions and five positive interactions, while the other contains five negative interactions and six positive interactions. According to MP-score, under a high α background, the former will be classified to be monochromatic, whereas the latter will not. It contradicts to the intuition that neither complex is monochromatic. In stringent data, cytosolic large ribosomal subunit, for instance, contains 5 positive interactions and 6 negative interactions, but it is absurdly identified as monochromatic complex (Additional File 3: Table S2).

So far, we have demonstrated that MP-score has several deficiencies, including background effect and functional design, and may not serve as an adequate measure to assess monochromaticity of clusters of genetic interactions. Therefore, developing an unbiased index is required.

### Genetic interactions within complexes present significant monochromatic tendency

We developed monochromatic index (MCI) to quantify the monochromatic tendency presented by all clusters of genetic interactions, not individual cluster (see Materials and Methods for details). Unlike MP-scores which determine the monochromaticity of each cluster, MCI calculates the overall monochromaticity directly. Note that a cluster of genetic interactions can be a set of interactions that belongs to a specific protein complex, or a collection of interactions between two complexes, depending on the research interest.

MCI is based on the following rationale. The consistency of types of genetic interactions within a cluster is measured by the ratio of difference between numbers of positive and negative interactions to the total number of interactions belonging to the cluster (denoted by β; see Materials and Methods). Then, MCI is calculated by a weighted mean of these β’s according to amounts of interactions of each cluster, reflecting the general belief that clusters containing more interactions are more reliable. To evaluate how significant the observed MCI was, we conducted a permutation test (Materials and Methods). Numbers of genetic interactions in each cluster were fixed, whereas the interactions were replaced by other genetic interactions which were randomly chosen from all screened pairs. The significance was assessed both by the proportion of null MCIs that are larger than the observed one and by a heuristic Z-score.

**Figure 3:**
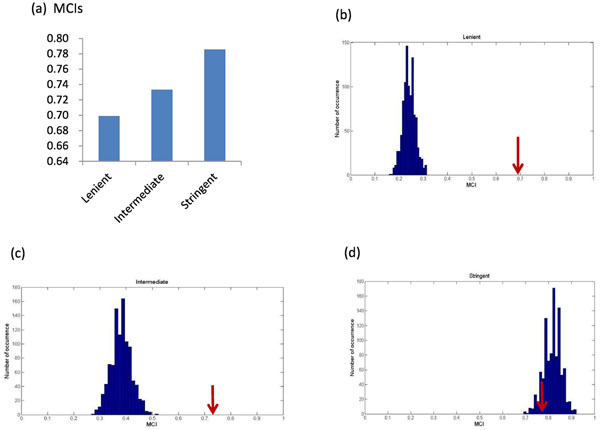
**Evaluate monochromaticity of within-complex interactions by MCI.** (a) MCIs of lenient, intermediate, and stringent cutoff data sets. MCI increases as the cutoff threshold becomes more stringent. (b-d) Distributions of null MCIs from 1000 random permutation in (b) lenient, (c) intermediate, and (d) stringent datasets. The observed MCIs are pointed by red arrows. Monochromaticity is significant in both lenient and intermediate datasets, but is not significant in stringent dataset. Our results show that MCI properly reflects the background information.

We then applied MCI to the three different cutoff datasets to investigate the monochromaticity of genetic interactions within protein complexes. MCI increases as the cutoff threshold becomes more stringent (Figure 3a), which is expected by the fact that the more stringent the data set is, the more biased the background will be. The significance of monochromaticity can be evaluated by comparing the observed MCI to null MCIs. The lenient dataset showed strong evidence supporting the existence of monochromaticity in within-complex genetic interactions (Figure 3b), whereas, although with the highest MCI, monochromaticity is not corroborated by the stringent dataset (Figure 3d), which is expected due to the highly biased background. This result demonstrated that MCI not only provided a quantitative assessment of monochromaticity for clusters of genetic interactions, but also reflected the background effect properly.

Based on lenient dataset, the previous study of Baryshnikova et al. suggested that genetic interactions in protein complexes are monochromatic [9]. Our result also revealed that within-complex interactions present significant monochromatic tendency. In addition, applying MCI to Collins’ dataset [13], we still had the same findings. However, MP-score fails to detect the monochromaticity in Collins’ dataset (Additional File 4: Table S3). The degrees of monochromaticity of all within-complex clusters are listed in Additional File 5: Table S4.

### Genetic interactions between protein complexes also present significant monochromatic tendency

After confirming that subunits of protein complexes connect to each other monochromatically [9], we were interested in investigating the monochromatic tendency of genetic interactions between protein complexes. We use the lenient dataset for the following analysis.

**Figure 4:**
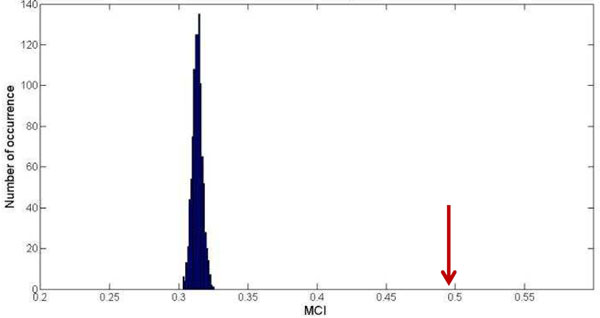
**Monochromaticity of between-complex interactions.** The histogram shows the distribution of null MCIs from 1000 random permutation in lenient dataset. The observed values of MCI for between-complex clusters are indicated by red arrow.

A genetic interaction is defined as a between-complex interaction if the two interacting genes belong to two different protein complexes. A cluster is a set of between-complex interactions connecting two specific protein complexes. Here we identified 32,329 between-complex interactions, which are separated into 7,673 clusters. The 5,432 clusters, each containing at least two genetic interactions, were used to evaluate the monochromaticity between protein complexes. The MCI is 0.49 which is lower than the MCI of within-complex interactions. The level of monochromaticity between protein complexes is not as high as within protein complexes; however, compared with randomly generated clusters, between-complex interactions still present a significant monochromatic tendency (Figure 4).

The result that significant monochromatic tendency exists in genetic interactions between protein complexes has never, to our knowledge, been proposed before; and it cannot be detected by MP-score (Additional File 6: Table S5). The results of all between-complex clusters can be found in Additional File 7: Table S6.

### Different cellular subsystems manifest distinct monochromatic patterns

To gain more insight into genetic interactions, we compared within-complex and between-complex interactions of different cellular subsystems. Since cellular functions can be roughly classified into three categories, informational transduction, genetic expression, and material and energy process [28], three cellular subsystems were chosen, signaling, transcription and translation, and metabolic networks. Gene lists of the three subsystems were obtained from Gene Ontology annotations [26], with GO:002352, GO:0010476, and GO:0008152 standing for signaling, gene expression and metabolic process, respectively. All genes in descendant GO terms were up-propagated to the three chosen terms. A protein complex is assigned to a subsystem if all the member genes of the protein complex belong to this subsystem.

We identified eight complexes for the signaling system, 26 for the transcription and translation system, and 30 for the metabolic system. Among the eight complexes in the signaling system, only two of them contain two or more genetic interactions and can be used for monochromatic analysis; this is attributed to the random choice of genes in the SGA experiment conducted by Costanzo et al [10]. Therefore, in the following analysis, we only considered the transcription and translation system, and the metabolic system.

Protein complexes in metabolic system exhibit highly monochromatic tendency in within-complex interactions, with MCI being 0.82 (Table 2). In fact, 77 % (10/13) of protein complexes presented purely positive (5) or purely negative (5). For instance, there are 15 genetic interactions identified in oligosaccharyltransferase (OST), and all are negative interactions (Additional File 8: Figure S2a). This result is consistent with the previous reports that redundancy of functions exists between subunits of OST [29,30]. Compared with the highly monochromaticity of within-complex interactions, between-complex interactions in metabolic system presented a modest level of monochromaticity with a MCI of 0.6 (Table 2). Among all 139 clusters, 67 clusters (48%) are purely monochromatic, with 45 being purely negative and 22 being purely positive. The largest cluster appears between alpha-1,6-mannosyltransferase complex and oligosaccharyltransferase complex, which contains 21 genetic interactions, and most are negative (20/21) (Additional File 8: Figure S2b), which suggests that these two complexes function in parallel, but compensatory, biological pathways.

**Table 2:** Summary of MCI in metabolic network.

	Within complexes	Between complexes
MCI	0.8182	0.6044
p-value	< 0.001	< 0.001
Null MCI summary	Mean: 0.3448 Std: 0.0861	Mean: 0.4227 Std: 0.0295
Z-score	5.4956	6.1712

MCIs of within-complex interactions and between-complex interactions are given. The p-value is the proportion of null MCIs that are greater than the observed one. We used random permutation to produce the 1000 null MCIs (Materials and methods).

Similar to what was observed in the metabolic system, genetic interactions connecting subunits of protein complexes in transcription and translation system exhibited a highly monochromatic propensity (Table 3). Nine purely monochromatic protein complexes were identified out of all 15 complexes, with 2 purely positive complexes and 7 purely negative ones. There are several large complexes in this system, such as cytosolic large ribosomal subunit complex (64 members), Srb-mediator complex (24 members), and Rpd3L complex (12 members). Although these larger complexes did not manifest purely monochromatic connections, they exhibited high levels of monochromaticity. Cytosolic large ribosomal subunit complex, for instance, contains 163 within-complex interactions, among which positive genetic interactions were more than 7 times of negative ones (144 to 19) (Additional File 9: Figure S3a).

**Table 3:** Summary of MCI in transcription and translation system.

	Within complexes	Between complexes
MCI	0.7594	0.4660
p-value	< 0.001	< 0.001
Null MCI summary	Mean: 0.1539 Std: 0.0427	Mean: 0.2463 Std: 0.0150
Z-score	14.1835	14.6793

MCIs of within-complex interactions and between-complex interactions are given. The p-value is the proportion of null MCIs that are greater than the observed one. We used random permutation to produce the 1000 null MCIs (Materials and methods).

In contrast to the high level of monochromaticity in within-complex interactions, protein complexes in transcription and translation system interacted to each other in a relatively low-monochromaticity manner, with a MCI of 0.47 (Table 3). Only 33% (68/209) of between-complex clusters were purely monochromatic.

**Figure 5:**
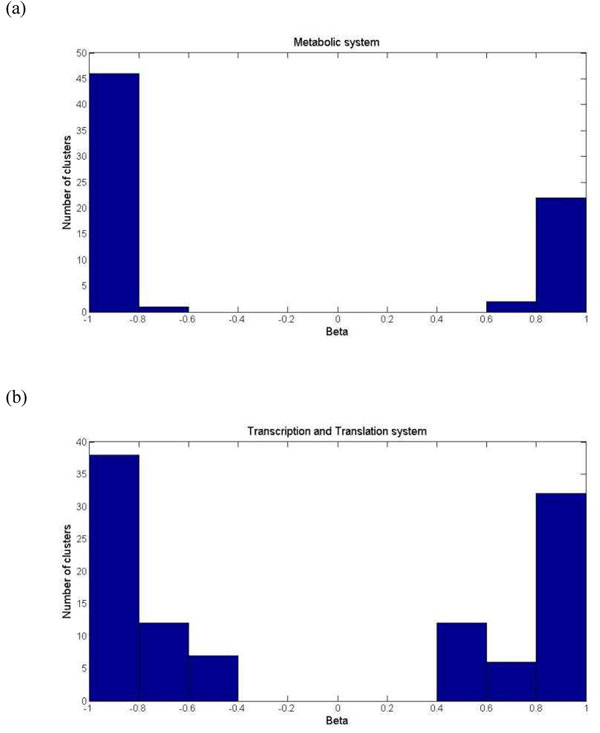
**Strategies adopted by different cellular subsystems.** We analyzed between-complex interactions in different cellular subsystems. Only highly monochromatic clusters were retained (Materials and methods). More negatively monochromatic clusters than the positive ones were found in metabolic system (a), while the numbers of positive and negative interactions were similar in transcription and translation system (b).

In addition to the level of monochromaticity in metabolic and transcription and translation systems, we further investigate the type of monochromaticity (positive or negative) which was dominant between protein complexes in these two systems. We identified highly monochromatic clusters of which the associated | *β* |’s were larger than the observed MCI (Materials and Methods). Then we calculated the distributions of these highly monochromatic clusters in the two cellular subsystems (Figure 5). Protein complexes interacted with each other in distinct patterns in these two systems. In metabolic system, negative connections between complexes were approximate two times than positive connections (47 to 24), whereas proportions of positive and negative between-complex clusters were similar in transcription and translation system, with a ratio of 50/57. This observation suggested that metabolic system tends to adopt a compensatory organization in functional relationship between protein complexes, while the proportion of complexes that work in concert is higher in transcription and translation system than in metabolic system. This result responses to the known fact that metabolic network is robust, with functional redundancy and compensatory metabolic fluxes [31,32]. It also suggested that transcription and translation system may be more vulnerable to complex dysfunctions than metabolic system due to the relatively lower proportion of compensatory relationships between protein complexes.

**Figure 6:**
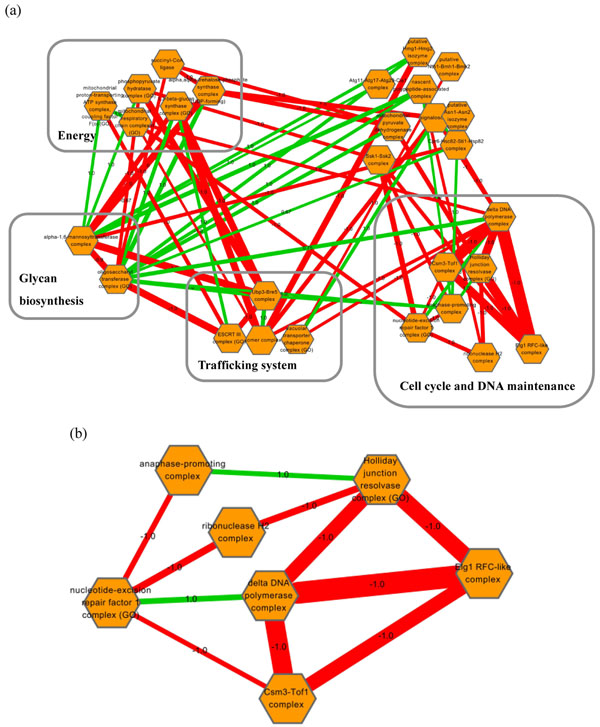
**The relationship between protein complexes in metabolic system.** (a) We plot several highly monochromatic between-complex clusters in metabolic system. The complexes were grouped together according to their functions. The unlabeled module includes complexes associated to processes such as autophagy, trehalose biosynthesis, and ergosterol biosynthetic process. (b) The enlargement of cell cycle and DNA maintenance. Each node represents a protein complex. Green edges: positive interactions. Red edges: negative interactions. Each edge is labeled with its β. The edge size is proportional to the mean of the genetic interactions.

**Figure 7:**
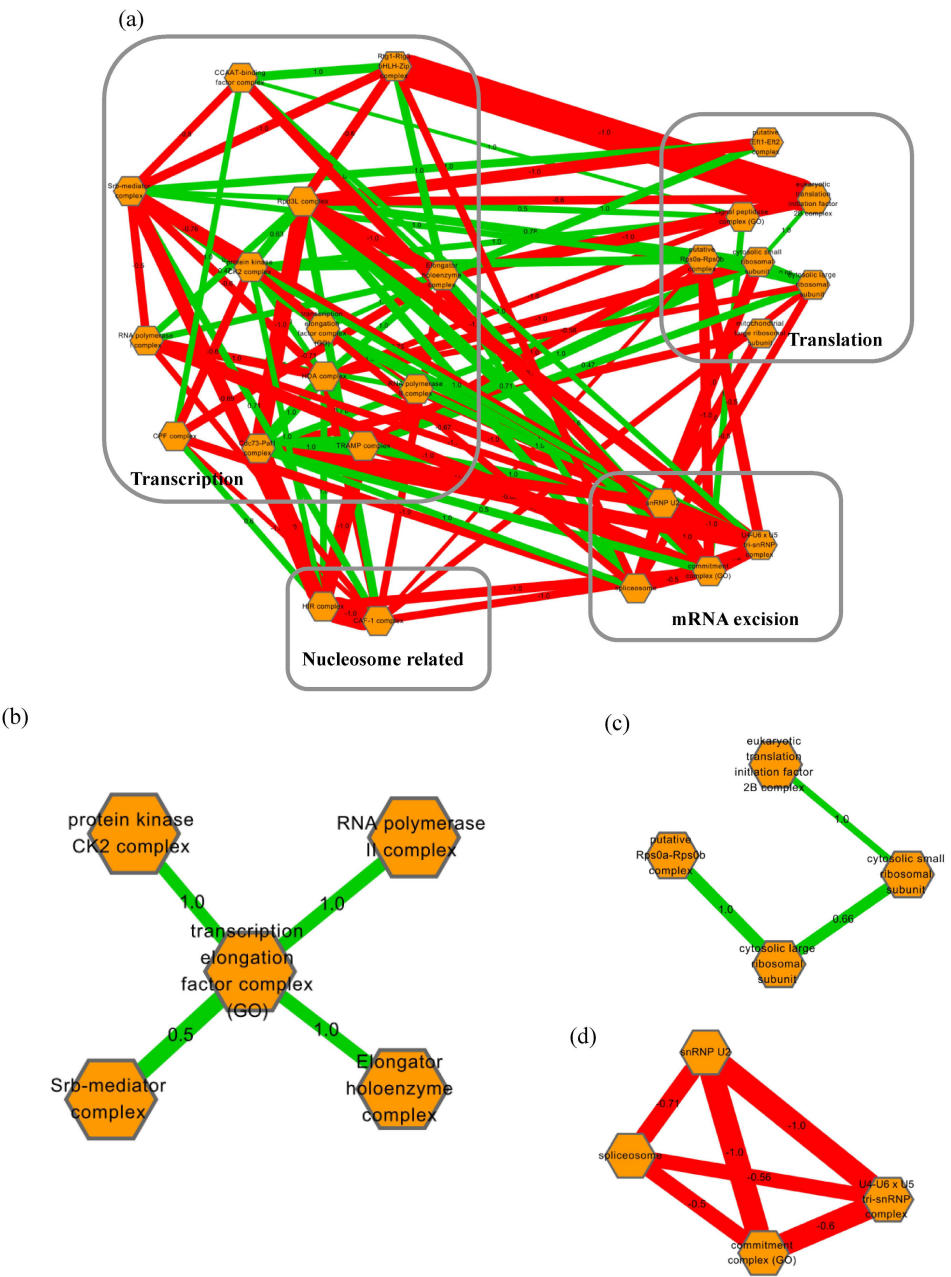
**The relationship between protein complexes in transcription and translation system.** Several parts of highly monochromatic between-complex clusters in transcription and translation system. Relation between transcription and translation processes (a). Enlargements of transcription (b), translation (c), and spliceosome (d).

In order to gain insights into the relations between protein complexes in metabolic, and transcription and translation systems, we plotted a few highly monochromatic between-complex clusters (Figure 6, Figure 7). For the metabolic system, there were more negative interactions connecting protein complexes (Figure 6). It might be worth noting that a large proportion (58%) of positive interactions are related to glycan biosynthesis which includes oligosaccharyl transferase complex and alpha-1,6-mannosyltransferase complex. This observation indicated that dysfunction of this process may compromise many other biological processes in the metabolic system. A closer scrutiny of cell cycle and DNA maintenance related complexes revealed that Holliday junction resolvase complex is linked to anaphase-promoting complex by a positive interaction, which reflects their functional order during meiosis (Figure 6b). In addition, Elg1 RFC-like complex, delta DNA polymerase complex, and Csm3-Tof1 complex strongly interacted to each other negatively, suggesting functional redundancy or compensation between these complexes.

On the other hand, transcription and translation system presents higher proportion of positive interaction than the metabolic system (Figure 7a). In transcription module, Srb-mediator complex which is required for transcriptional activation, protein kinase CK2 complex which phosphorylates RNA polymerases, RNA polymerase II complex, and Elongator holoenzyme complex are all connected to transcription elongation factor complex by positive interactions, demonstrating they function in order during transcription process (Figure 7b). As for translation process, cytosolic small ribosomal subunit, cytosolic large ribosomal subunit and eukaryotic translation initiation factor 2B complex are linked by positive interactions, indicating that these complexes have to work together to accomplish translation process (Figure 7c). Another interesting observation was that spliceosome-related complexes form a negative clique, which suggests that these complexes can compensate each other (Figure 7d). Moreover, the relationships between modules can also be investigated through the MCI-generated networks. For example, many negative connections were found between the mRNA excision module and the translation module, indicating that dysfunction of the mRNA excision module will aggravate the effect of the loss-function of the translation module (Additional File 10: Figure S4).

These results demonstrated that MCI can quantitatively measure the levels of monochromaticity, facilitating comparing the monochromatic tendencies in different cellular subsystems. In addition, MCI also unveils strategies used by different cellular subsystems to coordinate protein complexes, and provides insights into the features of each cellular subsystem. Furthermore, through the network of genetic interactions between protein complexes, the functional relationships between protein complexes can easily be studied; features of modules can be revealed; and MCI also provides a step toward studying the functional relationships between modules.

## Conclusion

Genetic interactions have been proved to be a powerful tool to study the relation between genotypes and phenotypes [9-13,17,19]. However, due to the underlying complexity of the genetic interaction network, it is still far from being able to precisely predict the operations and responses of a cell solely by its genetic configuration.

Monochromaticity serves as a possible guideline to cluster the complicated network into functional modules, which often represent protein complexes or biological pathways [22,24]; it also assists us to understand how these genes cooperate to determine phenotypes. Unfortunately, no appropriate measurement can quantify the level of monochromatic patterns, and thus limits the possibility of integrating monochromaticity with other quantitative characteristics to further explore the genetic landscape.

This study proposes a measure called monochromatic index (MCI) which is able to quantitatively evaluate the extent of monochromaticity of clusters of genetic interactions. This index not only amends the deficiencies of the previously proposed MP-score, but also provides a significant level for the observed quantity. By analyzing the genetic interaction data provided by Costanzo et al. [10] with MCI, we confirmed the previously described monochromatic pattern within protein complexes. Moreover, we also found that connections between protein complexes also presented a significant tendency of monochromaticity, which has never been proposed before.

Furthermore, MCI also reveal various genetic landscapes in different cellular subsystems. To be more specific, levels of within-complex (between-complex) monochromaticity of different cellular subsystems can be measured by MCI. In addition, MCI also unveils how protein complexes are coordinated in different cellular subsystems. Here, we study the metabolic system, and transcription and translation system. The results show that protein complexes participating in the metabolic system connect to each other mainly through negative interactions, while similar proportions of positive and negative interactions are identified between protein complexes in transcription and translation system. This result not only responses to previous reports that robustness of metabolic system can be partly explained by paralogue redundancy and compensatory metabolic fluxes [31,32], but also hypothesizes transcription and translation system adopts much higher proportion of protein complexes that have to work in concert or in serial pathways. It helps us to gain insight into strategies utilized to coordinate protein complexes by different cellular subsystems in terms of genetic interactions.

With MCI, a genetic interaction network of protein complexes is easily charted. It facilitates investigating the functional relationships between protein complexes, the features of modules, and the relationships between modules. It also helps researchers to design experiments to study protein complexes of interest, accelerating the pace toward understanding the relation between genotypes and phenotypes.

MCI evaluates the level of monochromaticity from an "edge-based" point of view. That is, only numbers of positive and negative genetic interactions in clusters are considered. Monochromaticity is interpreted as how dominant – in the sense of amount – a particular type (positive or negative) of genetic interaction is in a cluster. On the other hand, monochromaticity can also be comprehended by considering strengths of genetic interactions between members in a cluster. Therefore, we also proposed strength-based monochromatic index (sMCI) to explore monochromaticity (see Materials and Methods). The results were similar to those obtained by MCI (Additional Files 11, 12, 13: Figure S5-S7). Within-complex interactions were reported to have sMCI equal to 0.71, while the value is 0.57 for between-complex interactions. Besides, more negative connections between protein complexes were found in the metabolic system (25 positive and 51 negative), whereas transcription and translation system had more balanced proportions (40% positive and 60% negative). These observations illustrated that monochromaticity can be evaluated in terms of interaction amounts or strengths.

Although the most comprehensive genetic interaction data was adopted in this work, it has to be admitted that a large amount of interaction pairs have not been investigated in the experiment [10], which may significantly affect our results. Some protein complexes were not considered because of having no or few genetic interactions. Even protein complexes that were considered to be highly monochromatic may suffer a bias caused by incompleteness of exploration of all possible genetic interactions. To account for the effect of incompleteness on monochromaticity, we weighted each protein complex according to its number of genetic interactions that have been screened in the experiment when calculating MCI. In other words, we assumed that the more interactions a protein complex contains, the more reliable it is for evaluating monochromaticity. In addition, the study of cellular subsystems was also restricted to available genetic interactions. Only the metabolic system and the transcription and translation system contain enough protein complexes, and thus were analyzed. As more genetic interactions are screened, we expect that more solid conclusions can be drawn and strategies adopted by different cellular subsystems can be illustrated.

Modularity has been demonstrated to be a characteristic feature of cellular networks [26,28]. Hierarchical agglomerative clustering is widely adopted when investigating the modularity of a given system [10,13,17]. However, when facing an unknown network, the number of clusters is hard to determine. Although unsupervised learning can be utilized, it is often difficult to give the results a biological interpretation. We expect that monochromaticity can serve as an auxiliary guideline to define borders between functional modules. For example, a network can be divided in such a way that members in each group interact monochromatically and monochromaticity between clusters reaches a high value. We believe that clearly defined functional modules can serve as the first step toward illustrating the relationships between genotypes and phenotypes.

## Materials and methods

### Genetic interaction data

The dataset used in this study comes from the most comprehensive genetic interaction experiment conducted in *Saccharomyces cerevisiae*[10]. The Synthetic Genetic Array technique (SGA) was adopted with 1,711 query genes and 3,885 array genes spanning all biological processes, resulting in 6,651,120 interaction pairs. Single mutant fitness (SMF) of each gene and double mutant fitness (DMF) of each gene pair are available online, and these two kinds of values were used for further analysis in this study.

The genetic interaction scores (ε) are also available online. There are three sets of genetic interaction scores with different cutoff strengths — that is, lenient, intermediate and stringent cutoffs. Lenient cutoff is defined by p-value smaller than 0.05, whereas intermediate cutoff is defined by p-value < 0.05 and |ε| > 0.08. Stringent dataset contains those genetic interaction scores which meet one of the conditions — that is, ε > 0.16 and p-value < 0.05 or ε < -0.12 and p-value < 0.05.

### Protein complexes data

Consensus protein complex data in Benschop’s study was adopted [27]. We obtained the gene list of 518 protein complexes from the supplementary data of their study. The 518 protein complexes consist of 235 complexes that were confirmed in literature, 174 predicted complexes, and 109 protein complexes defined by GO terms.

### Monochromatic purity score

In previous work [9], the monochromaticity of interactions within a complex was evaluated by monochromatic purity score (MP-score) which was defined as:

where,

N_i_ = number of screened pairs within complex i.

N_total_ = number of total screened pairs.

C_i_ = member genes of complex i.

For a protein complex of which the interactions are all positive, the MP score is +1, whereas for a protein complex of which the interactions are all negative, the MP score is -1. For a protein complex with a MP score of zero, the protein complex has the same ratio of positive interactions to negative ones as that of background. A protein complex is considered to be monochromatic if it satisfies |*MP*(*C_i_*)| > 0.5.

### Binomial test for the monochromatic trend of complexes

A binomial test was adopted to examine the hypothesis that genetic interactions within protein complexes are mostly monochromatic. For the null hypothesis, we assumed that a protein complex has the same probability to be, or not to be, monochromatic. Hence, the number of monochromatic complexes within a complex population with size n could follow the binomial distribution: *Bin*(*n*, 0.5). The significance of monochromaticity was assessed by:

where,

*n* = total number of complexes in the population.

*x* = number of complexes that are identified to be monochromatic.

### Monochromatic index (MCI) and strength-based monochromatic index (sMCI)

We developed a measure called monochromatic index (MCI) to evaluate the extent of monochromaticity of clusters of genetic interactions. Clusters containing at least two interactions are retained in the population for calculating the MCI. MCI is defined as:

where,

C is the population of genetic interaction clusters.

N_total,i_ is the number of total genetic interactions in cluster i.

N_positive,i_ is the number of positive genetic interactions in cluster i.

N_negative,i_ is the number of negative genetic interactions in cluster i.

The quantity, , measures the monochromaticity of genetic interactions in a cluster. When | *β_i_* | = 1, the cluster is purely monochromatic; when |*β_i_* | = 0, the numbers of positive interactions and negative interactions in the cluster are the same. MCI is the mean of this quantity weighted by number of interactions in the corresponding cluster, reflecting the faith that clusters with more interactions have stronger reliability. MCI ranges from 0, which indicates the equal numbers of positive and negative genetic interactions, to 1, which indicates pure positive or negative interactions. A cluster of which the associated | *β_i_* | is greater than the MCI is considered as a highly monochromatic cluster.

In a similar rationale, strength-based monochromatic index (sMCI) is defined by:

where,

, *where ε_j_ is the strength of genetic interaction j.*

C is the population of genetic interaction clusters.

N_total,i_ is the number of total genetic interactions in cluster i.

N_positive,i_ is the number of positive genetic interactions in cluster i.

N_negative,i_ is the numberof negative genetic interactions in cluster i.

### Evaluate the significance of MCI and sMCI

We use permutation test to assess the significance of MCI and sMCI. In the permutation test, genetic interactions in each cluster are replaced by genetic interactions randomly chosen from all screened interactions while keeping the total number of interactions in each cluster fixed. A distribution of null MCI (sMCI) is then obtained, to which the MCI (sMCI) of the population of clusters is compared. Z-score is adopted to give a quantitative measure of the significance:

where,

µ is the mean of null MCIs.

s is the standard deveation of null MCIs.

## Competing interests

The authors declare that they have no competing interests.

## Authors' contributions

CHH and TYW developed the statistical methods, carried out the statistical analysis, participated in the design of the study and drafted the manuscript. HTC participated in the design of the study. CYK and KCC conceived of the study, and participated in its design and coordination and helped to draft the manuscript. All authors read and approved the final manuscript.

## Supplementary Material

Additional File 1 **Table S1. Number of genetic interactions screened in the three data sets.** Number of positive and negative genetic interactions in the three data sets is showed. It can be seen that there is a bias toward negative genetic interactions as cutoff becomes more stringent. Click here for file

Additional File 2 **Figure S1. Proportion of positive and negative genetic interactions.** Positive and negative genetic interactions are showed with blue and red respectively. It can be seen that in the stringent cutoff dataset, the proportions of the positive and negative interactions are extremely unbalanced. Click here for file

Additional File 3 **Table S2. MP-score misjudges the monochromaticity of protein complexes under highly biased background.** In highly biased background, both golgi transport complex and cytosolic large ribosomal subunit are misjudged to be monochromatic by MP-score. Furthermore, the MP score of cytosolic large ribosomal subunit is higher than that of golgi transport complex, implicating that cytosolic large ribosomal subunit is more monochromatic than golgi transport complex. However, with the proportions of genetic interactions of these two protein complexes, it is obviously an absurd conclusion. Click here for file

Additional File 4 **Table S3. Analysis of Collins’ dataset by MCI and MP-score.** (a) MCI revealed that within-complex interactions present significant monochromatic tendency. However, (b) MP-score fails to detect the monochromaticity in Collins’ dataset. Click here for file

Additional File 5 Table S4. The degrees of monochromaticity of within-complex clusters Click here for file

Additional File 6 **Table S5. MP-score fails to detect significant monochromatic tendency in between-complex clusters.** MP-score assigns only 2,623 out of 5,432 clusters to be monochromatic; the associated p-value is 0.99 which does not give significance to the monochromaticity of between-complex clusters. Click here for file

Additional File 7 Table S6. The degrees of monochromaticity of between-complex clusters Click here for file

Additional File 8 **Figure S2. Examples of within-complex and between-complex clusters in metabolic system.** The figure contains (a) the within-complex interactions of OST and (b) between-complex interactions between OST and α-1,6-mannosyltransferase complex. Red edges indicate negative interactions. Green edges indicate positive interactions. The edge size is proportional to the strength of genetic interactions which are labeled on edges. Click here for file

Additional File 9 **Figure S3. Examples of within-complex and between-complex clusters in transcription and translation system.** The figure contains (a) the within-complex interactions of cytosolic large ribosomal subunit and (b) between-complex interactions between cytosolic large ribosomal subunit and cytosolic small ribosomal subunit. Red edges indicate negative interactions. Green edges indicate positive interactions. The edge size is proportional to the strength of genetic interactions which are labeled on edges. Click here for file

Additional File 10 **Figure S4. Examples of the functional relationships between modules.** The figure contains present the genetic interactions between the mRNA excision module and the translation module. Click here for file

Additional File 11 **Figure S5. Evaluate monochromaticity of within-complex interactions by sMCI.** (a) sMCIs of lenient, intermediate, and stringent cutoff data sets. (b) Distribution of null sMCIs from 1000 random permutation in lenient data set. The observed sMCI is pointed by red arrow. (c) Intermediate data set. (d) Stringent data set. Click here for file

Additional File 12 **Figure S6. Monochromaticity of between-complex interactions.** The histogram shows the distribution of null sMCIs from 1000 random permutation in lenient dataset. The observed value of sMCI for between-complex interactions is indicated by red arrow. Click here for file

Additional File 13 **Figure S7. Strategies adopted by different cellular subsystems.** We analyze between-complex interactions in different cellular subsystems. Only highly monochromatic clusters are remained (Materials and methods). More negatively monochromatic clusters the positive ones are found in metabolic network (a), while these numbers are similar in transcription and translation system (b). Click here for file
