# Supplementary material for: A quantitative analysis of monochromaticity in genetic interaction networks
Source: BMC Bioinformatics. 2011 Nov 30;12(Suppl 13):S16. doi: 10.1186/1471-2105-12-S13-S16 (PMC3278832; doi:10.1186/1471-2105-12-S13-S16)
Supplement: Additional File 1 — Table S1. Number of genetic interactions screened in the three data sets. Number of positive and negative genetic interactions in the three data sets is showed. It can be seen that there is a bias toward negative genetic interactions as cutoff becomes more stringent. [file 1471-2105-12-S13-S16-S1.pdf]

Supplementary data

Table S1. Number of genetic interactions screened in the three data sets. Number of positive and negative genetic interactions in the three data sets is showed. It can be seen that there is a bias toward negative genetic interactions as cutoff becomes more stringent.

	Positive interactions	Negative interactions
Lenient	355778	408076
Intermediate	67243	124066
Stringent	6956	67631
