# Supplementary material for: A quantitative analysis of monochromaticity in genetic interaction networks
Source: BMC Bioinformatics. 2011 Nov 30;12(Suppl 13):S16. doi: 10.1186/1471-2105-12-S13-S16 (PMC3278832; doi:10.1186/1471-2105-12-S13-S16)

Figure S1. Proportion of positive and negative genetic interactions. Positive and negative genetic interactions are showed with blue and red respectively. It can be seen that in the stringent cutoff dataset, the proportions of the positive and negative interactions are extremely unbalanced.

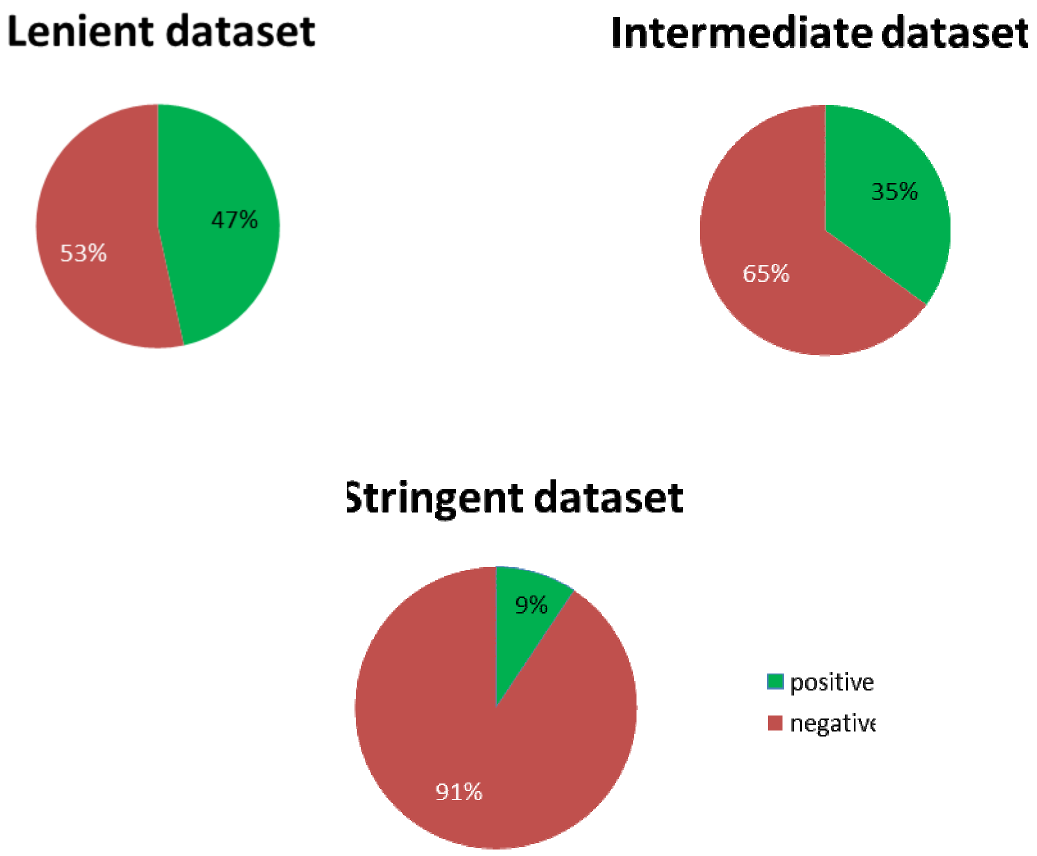

Supplement: Additional File 2 — Figure S1. Proportion of positive and negative genetic interactions. Positive and negative genetic interactions are showed with blue and red respectively. It can be seen that in the stringent cutoff dataset, the proportions of the positive and negative interactions are extremely unbalanced. [file 1471-2105-12-S13-S16-S2.pdf]
