# Supplementary material for: A quantitative analysis of monochromaticity in genetic interaction networks
Source: BMC Bioinformatics. 2011 Nov 30;12(Suppl 13):S16. doi: 10.1186/1471-2105-12-S13-S16 (PMC3278832; doi:10.1186/1471-2105-12-S13-S16)
Supplement: Additional File 3 — Table S2. MP-score misjudges the monochromaticity of protein complexes under highly biased background. In highly biased background, both golgi transport complex and cytosolic large ribosomal subunit are misjudged to be monochromatic by MP-score. Furthermore, the MP score of cytosolic large ribosomal subunit is higher than that of golgi transport complex, implicating that cytosolic large ribosomal subunit is more monochromatic than golgi transport complex. However, with the proportions of genetic interactions of these two protein complexes, it is obviously an absurd conclusion. [file 1471-2105-12-S13-S16-S3.pdf]

Table S2. MP-score misjudges the monochromaticity of protein complexes under highly biased background. In highly biased background, both golgi transport complex and cytosolic large ribosomal subunit are misjudged to be monochromatic by MP-score. Furthermore, the MP score of cytosolic large ribosomal subunit is higher than that of golgi transport complex, implicating that cytosolic large ribosomal subunit is more monochromatic than golgi transport complex. However, with the proportions of genetic interactions of these two protein complexes, it is obviously an absurd conclusion.

Complexes	Number of positive interactions	Number of negative interactions	MP score
COMA complex	0	7	-1
Golgi transport complex	4	7	2.90
Cytosolic large ribosomal subunit	5	6	3.87
