# Supplementary material for: A quantitative analysis of monochromaticity in genetic interaction networks
Source: BMC Bioinformatics. 2011 Nov 30;12(Suppl 13):S16. doi: 10.1186/1471-2105-12-S13-S16 (PMC3278832; doi:10.1186/1471-2105-12-S13-S16)
Supplement: Additional File 4 — Table S3. Analysis of Collins’ dataset by MCI and MP-score. (a) MCI revealed that within-complex interactions present significant monochromatic tendency. However, (b) MP-score fails to detect the monochromaticity in Collins’ dataset. [file 1471-2105-12-S13-S16-S4.pdf]

Table S3. Analysis of Collins' dataset by MCI and MP-score. MCI revealed that within-complex interactions present significant monochromatic tendency. However, MP-score fails to detect the monochromaticity in Collins' dataset.

(a)

MCI	0.3371
p-value	< 0.001

(b)

Total complex amount	Amount of positively monochromatic complexes	Amount of negatively monochromatic complexes	p-value for observing this proportion
56	17	11	0.5531
