# Supplementary material for: A quantitative analysis of monochromaticity in genetic interaction networks
Source: BMC Bioinformatics. 2011 Nov 30;12(Suppl 13):S16. doi: 10.1186/1471-2105-12-S13-S16 (PMC3278832; doi:10.1186/1471-2105-12-S13-S16)
Supplement: Additional File 6 — Table S5. MP-score fails to detect significant monochromatic tendency in between-complex clusters. MP-score assigns only 2,623 out of 5,432 clusters to be monochromatic; the associated p-value is 0.99 which does not give significance to the monochromaticity of between-complex clusters. [file 1471-2105-12-S13-S16-S6.pdf]

Table S5. MP-score fails to detect significant monochromatic tendency in between-complex clusters. MP-score assigns only 2,623 out of 5,432 clusters to be monochromatic; the associated p-value is 0.99 which does not give significance to the monochromaticity of between-complex clusters.

Total number of between-complex clusters	Number of positively monochromatic clusters	Number of negatively monochromatic clusters
5432	1386	1237
