# Supplementary material for: A quantitative analysis of monochromaticity in genetic interaction networks
Source: BMC Bioinformatics. 2011 Nov 30;12(Suppl 13):S16. doi: 10.1186/1471-2105-12-S13-S16 (PMC3278832; doi:10.1186/1471-2105-12-S13-S16)

Figure S2. Examples of within-complex and between-complex clusters in metabolic systems. The figure contains (a) the within-complex interactions of OST and (b) between-complex interactions between OST and alpha-1,6-mannosyltransferase complex. Red edges indicate negative interactions. Green edges indicate positive interactions. The edge size is proportional to the strength of genetic interactions which are labeled on edges.

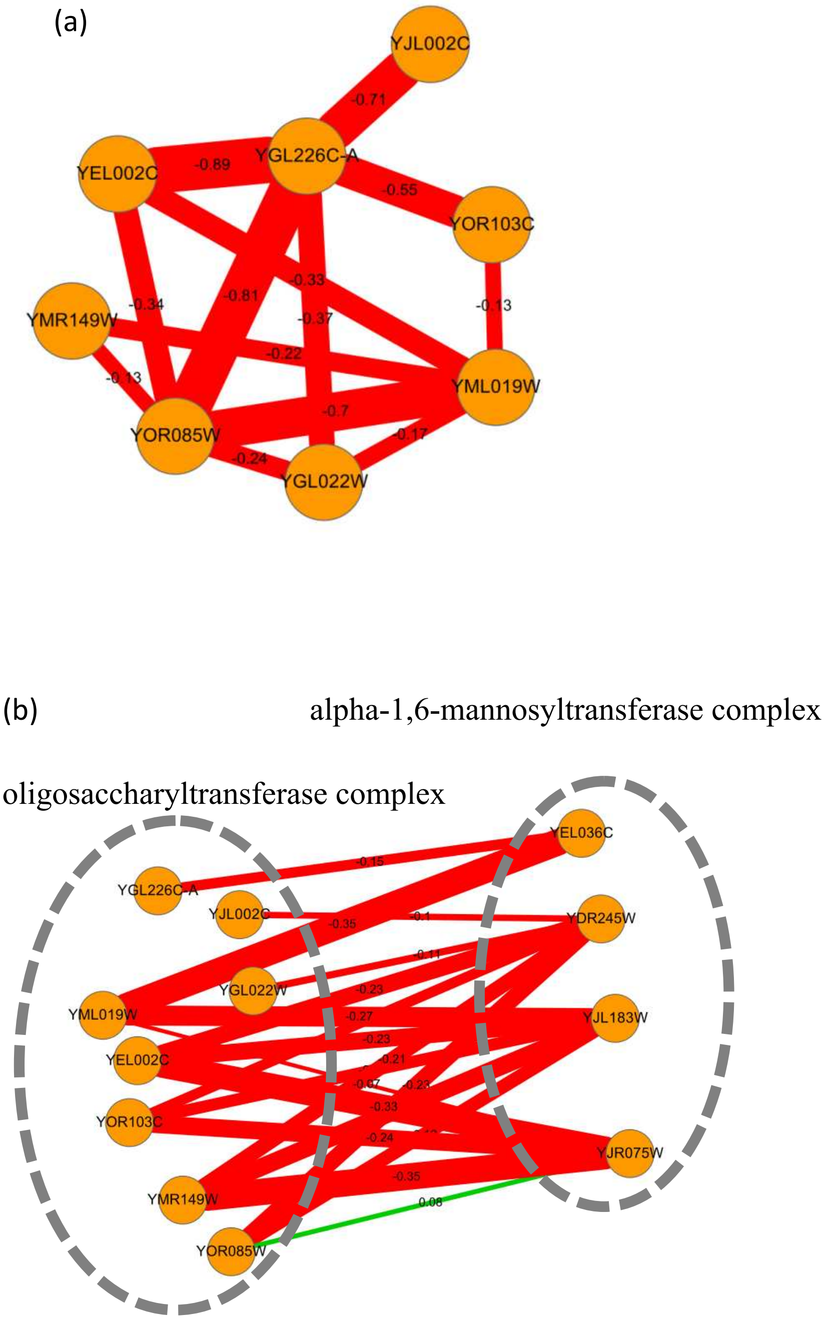

Supplement: Additional File 8 — Figure S2. Examples of within-complex and between-complex clusters in metabolic system. The figure contains (a) the within-complex interactions of OST and (b) between-complex interactions between OST and α-1,6-mannosyltransferase complex. Red edges indicate negative interactions. Green edges indicate positive interactions. The edge size is proportional to the strength of genetic interactions which are labeled on edges. [file 1471-2105-12-S13-S16-S8.pdf]
