# Supplementary material for: A quantitative analysis of monochromaticity in genetic interaction networks
Source: BMC Bioinformatics. 2011 Nov 30;12(Suppl 13):S16. doi: 10.1186/1471-2105-12-S13-S16 (PMC3278832; doi:10.1186/1471-2105-12-S13-S16)

Figure S3. Examples of within-complex and between-complex clusters in transcription and translation system. The figure contains (a) the within-complex interactions of cytosolic large ribosomal subunit and (b) between-complex interactions between cytosolic large ribosomal subunit and cytosolic small ribosomal subunit. Red edges indicate negative interactions. Green edges indicate positive interactions. The edge size is proportional to the strength of genetic interactions which are labeled on edges.

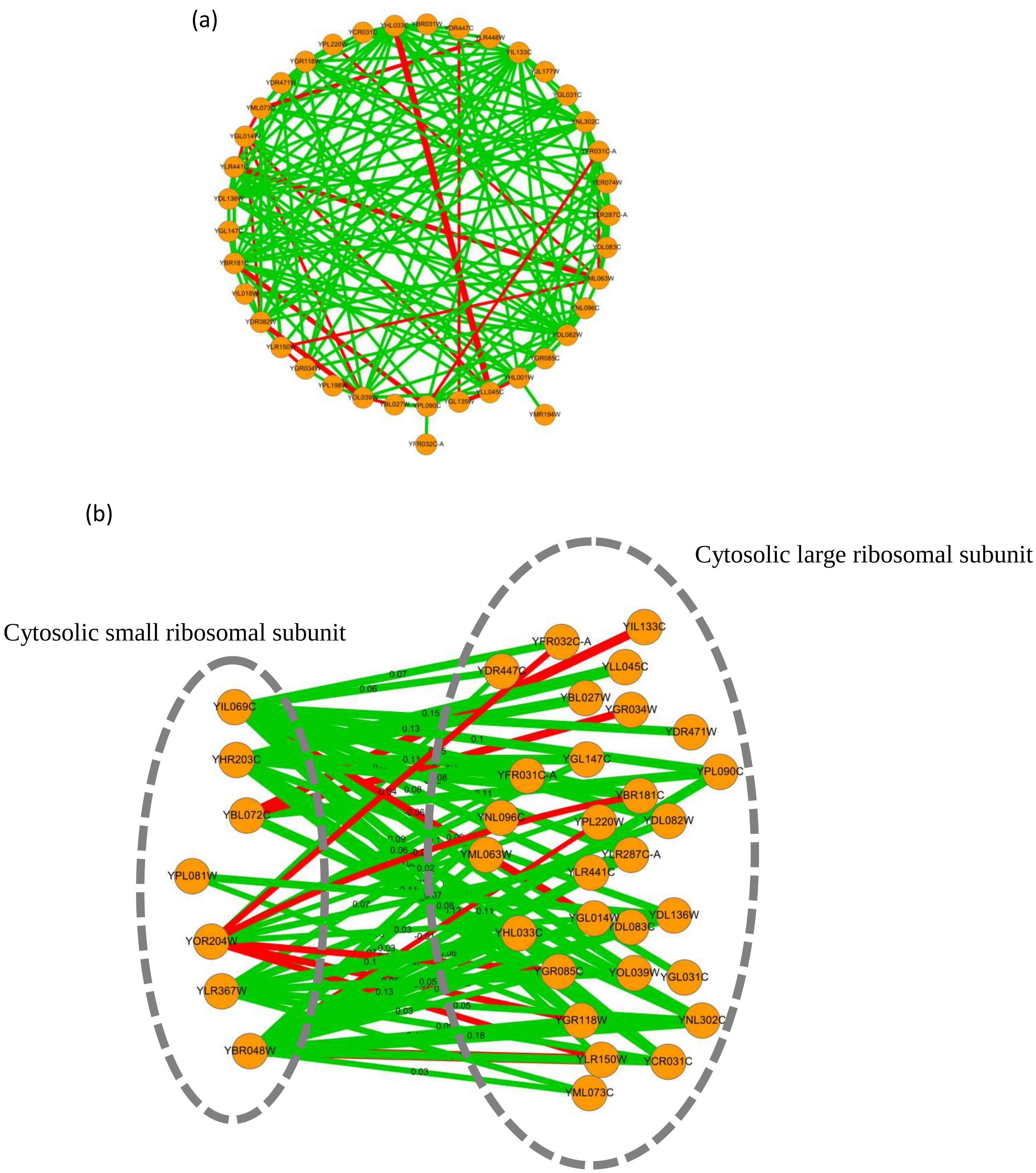

Supplement: Additional File 9 — Figure S3. Examples of within-complex and between-complex clusters in transcription and translation system. The figure contains (a) the within-complex interactions of cytosolic large ribosomal subunit and (b) between-complex interactions between cytosolic large ribosomal subunit and cytosolic small ribosomal subunit. Red edges indicate negative interactions. Green edges indicate positive interactions. The edge size is proportional to the strength of genetic interactions which are labeled on edges. [file 1471-2105-12-S13-S16-S9.pdf]
