# Supplementary material for: A quantitative analysis of monochromaticity in genetic interaction networks
Source: BMC Bioinformatics. 2011 Nov 30;12(Suppl 13):S16. doi: 10.1186/1471-2105-12-S13-S16 (PMC3278832; doi:10.1186/1471-2105-12-S13-S16)

Figure S4. Examples of the functional relationships between modules. The figure contains present the genetic interactions between the mRNA excision module and the translation module.

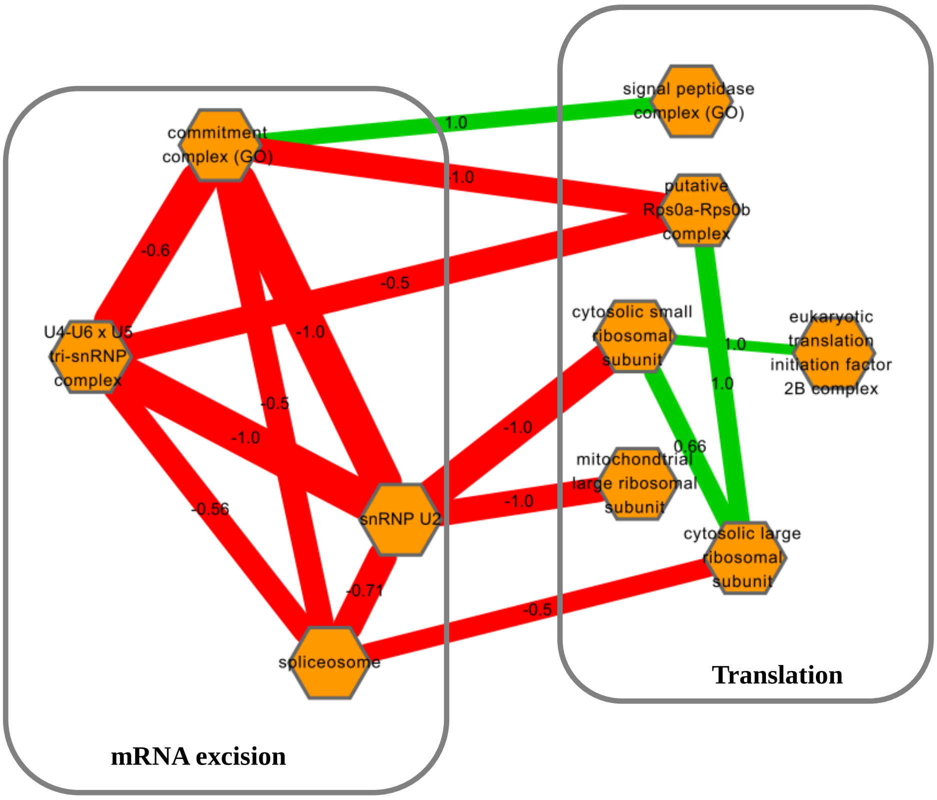

Supplement: Additional File 10 — Figure S4. Examples of the functional relationships between modules. The figure contains present the genetic interactions between the mRNA excision module and the translation module. [file 1471-2105-12-S13-S16-S10.pdf]
