# Supplementary material for: A quantitative analysis of monochromaticity in genetic interaction networks
Source: BMC Bioinformatics. 2011 Nov 30;12(Suppl 13):S16. doi: 10.1186/1471-2105-12-S13-S16 (PMC3278832; doi:10.1186/1471-2105-12-S13-S16)

Figure S5. Evaluate monochromaticity of within-complex interactions by sMCI.

(a) sMCIs of lenient, intermediate, and stringent cutoff data sets.

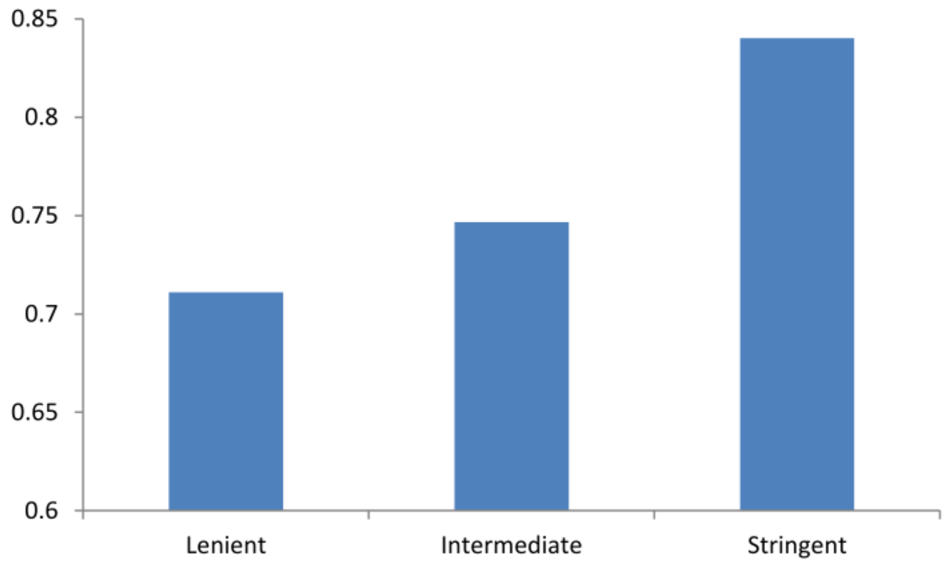

(b) Distribution of null sMCIs from 1000 random permutation in lenient data set. The observed sMCI is pointed by red arrow.

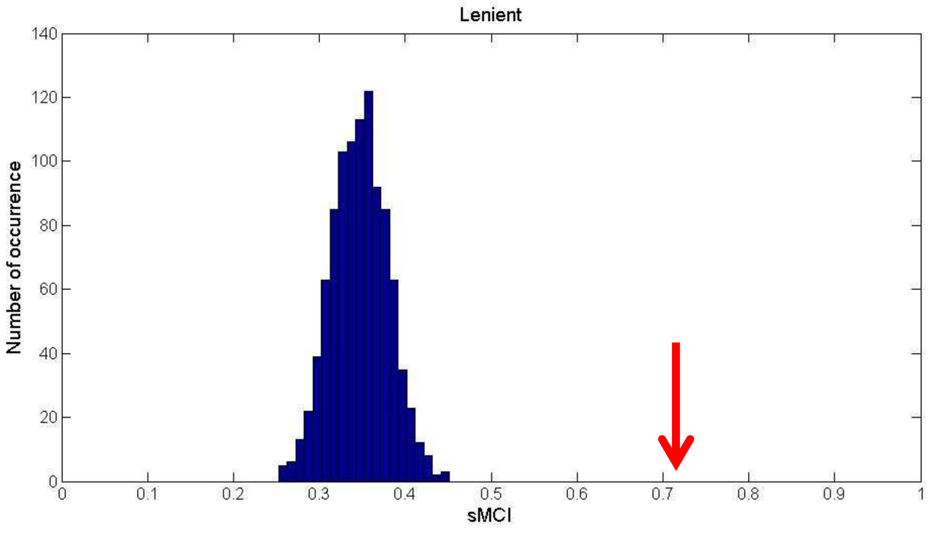

(c) Intermediate data set.

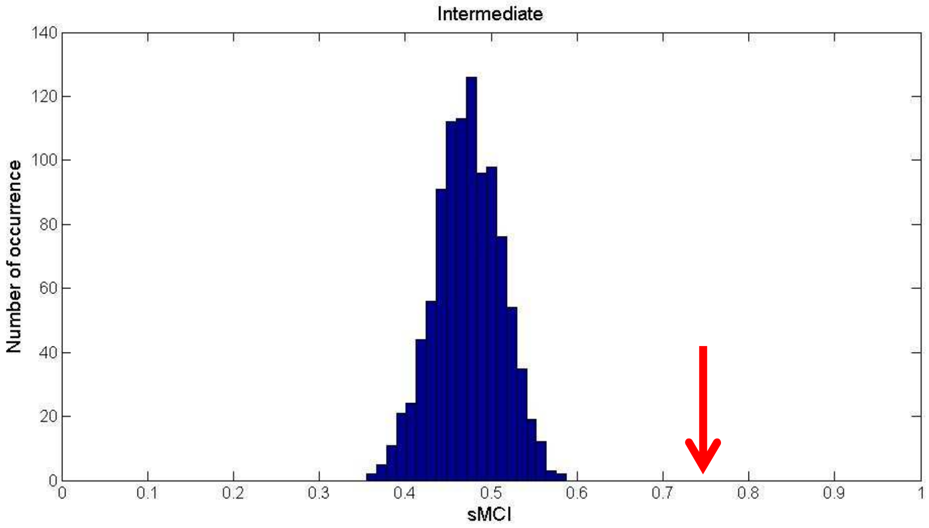

(d) Stringent data set.

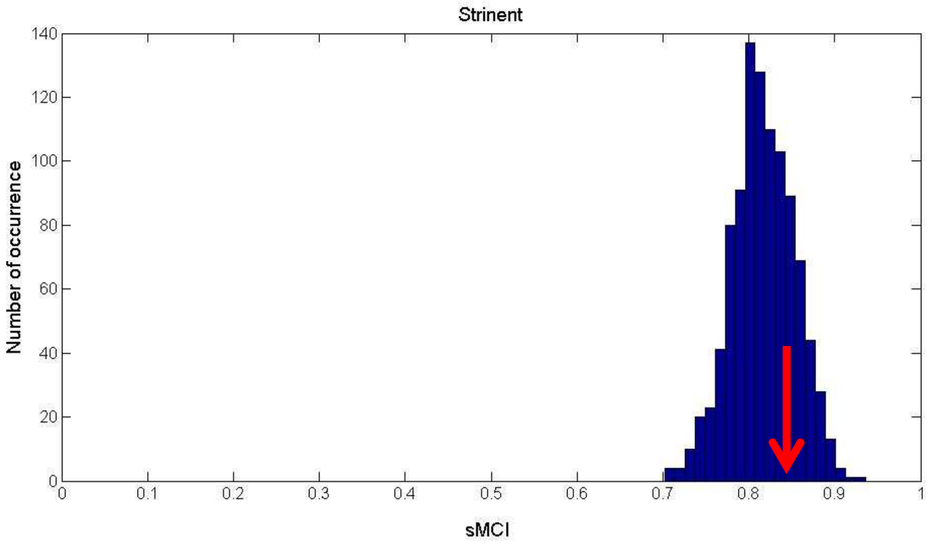

Supplement: Additional File 11 — Figure S5. Evaluate monochromaticity of within-complex interactions by sMCI. (a) sMCIs of lenient, intermediate, and stringent cutoff data sets. (b) Distribution of null sMCIs from 1000 random permutation in lenient data set. The observed sMCI is pointed by red arrow. (c) Intermediate data set. (d) Stringent data set. [file 1471-2105-12-S13-S16-S11.pdf]
