# Supplementary material for: A quantitative analysis of monochromaticity in genetic interaction networks
Source: BMC Bioinformatics. 2011 Nov 30;12(Suppl 13):S16. doi: 10.1186/1471-2105-12-S13-S16 (PMC3278832; doi:10.1186/1471-2105-12-S13-S16)

Figure S6. Monochromaticity of between-complex interactions. The histogram shows the distribution of null sMCIs from 1000 random permutation in lenient data set. The observed value of sMCI for between-complex interactions is indicated by red arrow.

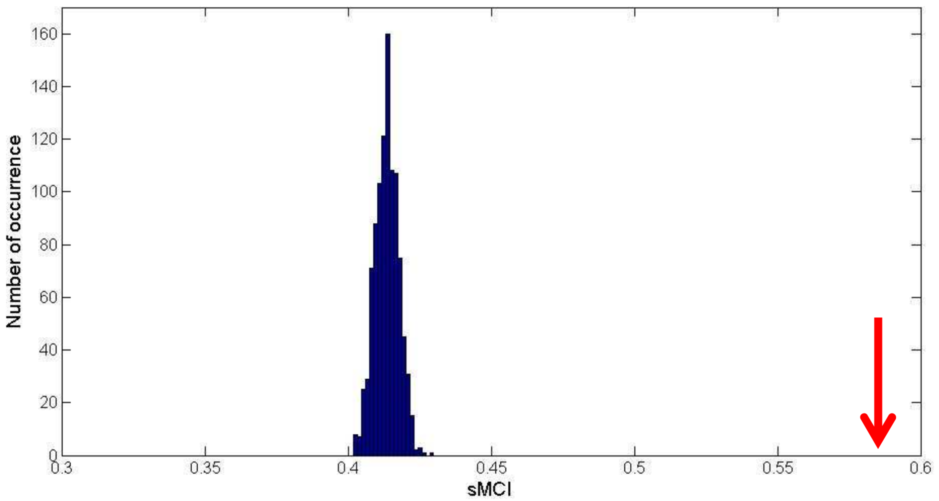

Supplement: Additional File 12 — Figure S6. Monochromaticity of between-complex interactions. The histogram shows the distribution of null sMCIs from 1000 random permutation in lenient dataset. The observed value of sMCI for between-complex interactions is indicated by red arrow. [file 1471-2105-12-S13-S16-S12.pdf]
