# Supplementary material for: A quantitative analysis of monochromaticity in genetic interaction networks
Source: BMC Bioinformatics. 2011 Nov 30;12(Suppl 13):S16. doi: 10.1186/1471-2105-12-S13-S16 (PMC3278832; doi:10.1186/1471-2105-12-S13-S16)

**Figure S7. Strategies adopted by different cellular subsystems.** We analyze between-complex interactions in different cellular subsystems. Only highly monochromatic clusters are remained (Methods). More negatively monochromatic clusters the positive ones are found in metabolic network (a), while these numbers are similar in transcription and translation system (b).

(a)

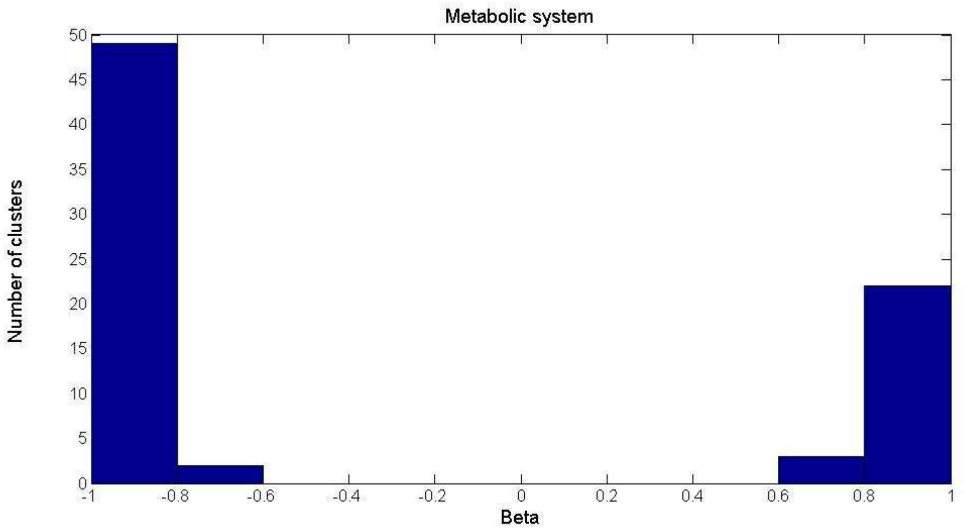


(b)

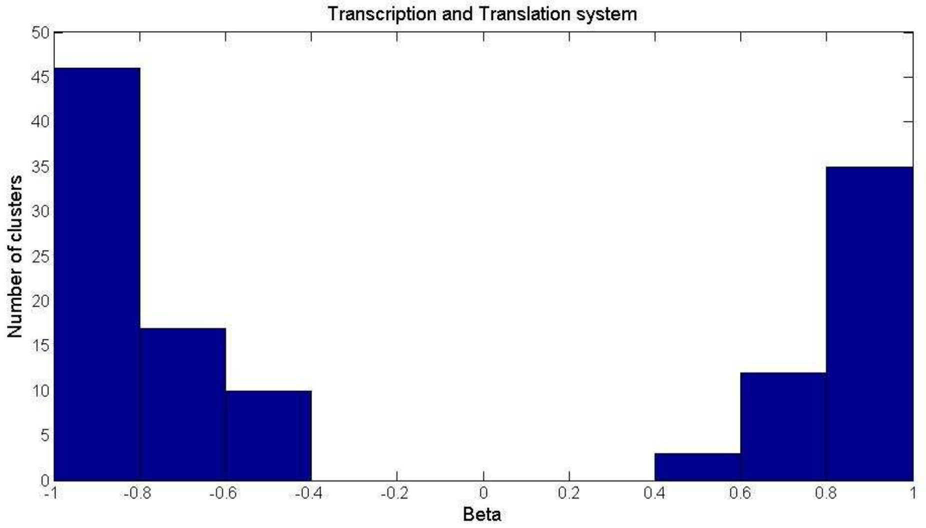

Supplement: Additional File 13 — Figure S7. Strategies adopted by different cellular subsystems. We analyze between-complex interactions in different cellular subsystems. Only highly monochromatic clusters are remained (Materials and methods). More negatively monochromatic clusters the positive ones are found in metabolic network (a), while these numbers are similar in transcription and translation system (b). [file 1471-2105-12-S13-S16-S13.pdf]
